# Curcumin Promotes the Clearance of *Listeria monocytogenes* both *In Vitro* and *In Vivo* by Reducing Listeriolysin O Oligomers

**DOI:** 10.3389/fimmu.2017.00574

**Published:** 2017-05-17

**Authors:** Xuan Zhou, Bing Zhang, Yumei Cui, Shuiye Chen, Zihao Teng, Gejin Lu, Jianfeng Wang, Xuming Deng

**Affiliations:** ^1^Center of Infection and Immunity, The First Hospital, Jilin University, Changchun, China; ^2^Key Laboratory of Zoonosis, Ministry of Education, College of Veterinary Medicine, Jilin University, Changchun, China

**Keywords:** anti-virulence, listeriolysin O, *Listeria monocytogenes*, molecular modeling, infection

## Abstract

The pore-forming toxin listeriolysin O (LLO), an essential virulence factor that is secreted by *Listeria monocytogenes* (*L. monocytogenes*), is responsible for bacterial breaching at the phagosomal membranes and subsequent release into the cytoplasm; it cannot be recognized by the host immune system. The vital role that LLO plays in bacterial pathogenicity and evading host immune clearance makes this virulence a promising target for addressing *L. monocytogenes* infection. In this study, we hypothesized that curcumin, a polyphenol derived from turmeric that could effectively inhibit LLO pore-forming activity, might be useful in the prevention or treatment of *L. monocytogenes* infection. Thus, the *in vitro* protective effects of curcumin against *L. monocytogenes* infection by targeting LLO were assessed via hemolytic activity assays, cytotoxicity tests, intracellular growth assays, and confocal microscopy. Our results revealed that treating infected macrophages with curcumin can lead to a decrease in LLO-mediated bacteria phagosomal escape and limit the intracellular growth of *L. monocytogenes*. Moreover, results from animal experiments show that this natural compound effectively increases protection against bacterial infection and helps the host to clear the invading pathogen completely from an animal model, establishing it as a potent antagonist of *L. monocytogenes*. The results from our molecular modeling and mutational analysis demonstrated that curcumin directly engages with domains 2 and 4 of LLO, thereby decreasing the hemolytic activity of LLO by influencing its oligomerization. Taken together, these results suggest that, as an antitoxin agent, curcumin can be further developed into a novel therapy against *L. monocytogenes* infections by targeting LLO.

## Introduction

The Gram-positive opportunistic bacterium *Listeria monocytogenes* (*L. monocytogenes*) is recognized as a zoonotic pathogen that is facultative and intracytosolic, and it causes listeriosis. Clinical manifestations of human *L. monocytogenes* infections range from non-symptomatic intestinal carriage and mild febrile gastroenteritis to meningitis, abortion, sepsis, and even fetal infections. Notably, despite the administration of current antibiotic therapy, which calls for high-dose antibiotics, the mortality rate of listeriosis can reach 30% among immunocompromised individuals, the elderly and pregnant women (1). With the continuing outbreak and prevalence of *L. monocytogenes* infections all over the world, and especially the antibiotic-resistant strains that have been isolated from humans and the environment, this bacterium is a major concern for public health (2–4).

*Listeria monocytogenes* is an invasive bacterium, and it expresses several virulence factors that are highly associated with cell invasion, intracellular bacterial survival, and cell-to-cell spreading. Following their internalization into target cells, including both phagocytic cells and diverse non-phagocytic cells, bacteria either are killed or end up escaping from the primary internalization vesicle into the cytoplasm (5). Once within the cytosol, the bacteria grow rapidly, and they utilize the host actin cytoskeleton by expressing a surface protein called ActA to form F-actin, which provides for bacterial motility and dissemination into neighboring cells. The pore-forming toxin listeriolysin O (LLO), in concert with PLCs (PI-PLC and PC-PLC), is the essential virulence factor that is required for destabilizing the vacuolar membrane and promoting the escape of the bacterium from the vesicle. The bacterium that resides in the host cell cytosol will undergo a novel round of proliferation. In this manner, bacteria are capable of completing their intracellular life cycle and avoiding exposure to the circulating components in the host immune system (6).

The early eradication of *L. monocytogenes* infection primarily relies on activated macrophages, neutrophils, natural killer (NK) cells, and T lymphocytes. Kupffer cells, the liver-resident macrophages, contribute primarily to trapping and destroying invasive bacteria by generating antimicrobial compounds (7). Busch et al. (8) have demonstrated that infecting mice with the indicated density of *L. monocytogenes* leads to the complete clearance of the bacteria from the spleen, which suggests that a low density of *L. monocytogenes* could lead to its complete clearance by the host immune system.

Throughout the intracellular life cycle, LLO, a member of the cholesterol-dependent cytolysin (CDC) family, is the critical virulence factor that is responsible for intracellular bacterial survival. This pore-forming toxin is secreted as a water-soluble monomer form that binds to a receptor on the organelles or the host plasma membrane, where the monomers oligomerize into a ring and through a sequential conformational change to form the membrane-inserting pore. The membrane insertion of LLO leads to a characteristic feature in which intracellular Ca^2+^ fluctuations lead to cell lysis from membrane perforation. Several studies have demonstrated that *L. monocytogenes* that lack LLO remain trapped within most cell types and are avirulent, and they display defects during intracellular bacterial growth in the host cell (9). Consistent with this finding, in comparison with wild-type strains, LLO-defective strains fail to cause mortality at a significantly lower bacterial burden in the murine model of systemic *L. monocytogenes* infection (10, 11). Taken together, these studies suggest that LLO can be a promising candidate for the development of a novel therapy against infections caused by *L. monocytogenes*.

In this work, we revealed a role for curcumin, a polyphenol derived from turmeric, which decreases LLO pore-forming activity by binding to the cleft between domain 2 and domain 4 of LLO, thereby interfering with LLO oligomerization. In this way, curcumin aids the host immune system in clearing bacteria by preventing them from escaping from the phagosomes. Additionally, we also observed that curcumin could strikingly inhibit the bacterial burden and protect mice from *L. monocytogenes* infection. Our results suggest that curcumin is a potent candidate against *L. monocytogenes* that acts by targeting LLO, and it may be a valuable alternative or adjunct to current antibiotic therapies.

## Materials and Methods

### Bacterial Growth and Reagents

The *L. monocytogenes* strains used in this study were the wild-type strains ATCC19115, EGD, and the LLO deletion mutant EGDΔ*hly*. Tryptic soy broth (TSB; Qingdao Hope Biol-Technology Co., Ltd.) was used to grow *L. monocytogenes* and *Escherichia coli* strains with shaking at 200 rpm and 37°C. Curcumin was purchased from the National Institute for the Control of Pharmaceutical and Biological Products (Beijing, China). When the experiment was finished, pathogens were sterilized by autoclave at 121°C for 30 min. Other chemical hazards were treated and disposed in accordance with the guidelines of Jilin University.

### Preparation of LLO and Its Mutants

The DNA sequence of LLO was PCR amplified from *L. monocytogenes* genomic DNA with the following primers: 5′-GCGCCATATGGATGCATCTGCATTCAATAAAG-3′ (forward) and 5′-GCGCCTCGAGTTCGATTGGATTATCTACTTTATTAC-3′ (reverse). The fragment was first cloned into a PET-21a expression vector and subsequently transformed into *E. coli* BL21 (DE3) cells for LLO expression.

The LLO mutagenesis was performed with a QuikChange Site-Directed Mutagenesis Kit. The primer pairs for these mutations were as follows: V100A forward, 5′ GATGGAAATGAATATATCGCGGTGGAGAAAAAGAAGAAATC 3′; V100A reverse, 5′ GATTTCTTCTTTTTCTCCACCGCGATATATTCATTTCCATC 3′; L503A forward, 5′ GATGACCGGAACTTACCAGCGGTGAAAAATAGAAATATCTCC 3′; and L503A reverse, 5′ GGAGATATTTCTATTTTTCACCGCTGGTAAGTTCCGGTCATC 3′.

Cultures of BL21 (DE3) cells harboring the vector that was cloned with the LLO, V100A, and L503A fragments were grown in TSB plus ampicillin at 37°C to an OD_600_ = 0.6. IPTG was then added to the TSB cultures at a final concentration of 0.5 mM with shaking for another 12 h at 16°C. The cells were then centrifuged and resuspended in an LLO lysis buffer (1× PBS, 1 mM DTT, and 1 mM PMSF) and subsequently broken by sonication. The supernatants were centrifuged at 4°C for 45 min, and the lysate was applied to a His-affinity column (GE Amersham). LLO and samples with specific mutations were eluted with 50 mM imidazole and concentrated in a storage buffer (35 mM Na_3_PO_4_, 125 mM NaCl, pH 5.5). The proteins were saved at −80°C for further studies.

### Minimal Inhibitory Concentration (MIC) Determination Assay

The MIC was defined as the lowest concentration of the drug at which no visible bacterial growth was observed, and the MIC value of curcumin against wild-type *L. monocytogenes* EGD was determined by the broth microdilution method as previously reported (12).

### Growth Curve Assay

For the growth curve assay, EGD was cultured in 120 ml of TSB at 37°C to an OD_600_ of 0.3 and aliquoted into six 50-ml Erlenmeyer flasks. Five of the cultures were supplemented with different concentrations of curcumin, which was pre-dissolved in DMSO at 0.25, 0.5, 1, 2, and 4 µg/ml. Then, the mixture samples were further grown at 37°C with shaking at 200 rpm, and the cell growth was monitored by reading the OD_600_ values every 30 min.

### Hemolytic Activity Assay

The hemolytic activity was measured to assess the protective effect of curcumin against LLO or its variants during mediated cells lysis. In brief, 1 µl of the purified LLO or its mutants were pre-incubated with different concentrations of curcumin at 37°C for 15 min. Then, 25 µl of sheep erythrocytes (5 × 10^6^ cells/ml) was added to each group. The mixture was incubated at 37°C for another 30 min, and then the erythrocytes were removed by centrifugation at 5,000 *g* for 1 min. The supernatant was measured at OD_543 nm_ by ultraviolet spectrophotometer. The curcumin-free cultures were used as 100% hemolysis controls, and the hemolysis was determined by comparing each sample to the control culture.

### Cytotoxicity Test

The protective effect of curcumin on *L. monocytogenes*-infected cells was determined by lactate dehydrogenase (LDH) release assays, and J774 macrophages were plated at a density of 2.0 × 10^4^ cells/well and grown overnight. The cells were infected with 200 µl of *L. monocytogenes* at moi = 5 with the indicated concentrations of curcumin, or they were treated with 10 ng of purified LLO, which was pre-incubated with different curcumin concentrations for 20 min at 37°C. After 5 h, the supernatants of each well were collected for analysis with a Cytotoxicity Detection Kit (LDH; Roche, Basel, Switzerland).

### Intracellular Growth Assays

The J774 macrophage-like cells were cultured in DMEM high glucose (1×; HyClone) and supplemented with 5% fetal bovine serum (Biological Industries). In this assay, the cells were seeded onto cover slips at 3 × 10^5^ cells/well on 13-mm cover slips with antibiotic-free medium overnight. The cells were incubated with 8 or 16 µg/ml curcumin and infected with bacteria at moi = 2.5 for 30 min. The cells were then washed five times with pre-warmed PBS and further incubated with 10 µg/ml gentamicin to kill extracellular bacteria. At arranged time points, the cover slips were placed in distilled water and vortexed violently for 5 min. The water containing the bacteria was then applied to solid TSB medium and cultured for 12 h.

### MD Simulations

The LLO structure was taken from the X-ray crystal structure in the Protein Data Bank (PDB) using PDB code 4CDB. The free protein obtained from the PDB (4CDB) was first equilibrated with a 100-ns molecular simulation of the solute, which was used for molecular docking with the inhibitor. The curcumin geometries were optimized at the B3LYP/6-31G* level with a Gaussian 03 program. The standard docking procedures for LLO with curcumin were performed with AutoDock4 (13, 14). Subsequently, a molecular dynamics simulation of the complex systems was performed using computational biology methods that have been described in detail in previous reports (15–17).

The Molecular Mechanics/Poisson–Boltzmann Surface Area (MM-PBSA) decomposition process was used to analyze the interaction between curcumin and each residue in the LLO binding site in Amber 10. The binding interaction of each curcumin–residue pair includes three terms, namely, the Van der Waals contribution (Δ*E*_vdw_), the electrostatic contribution (Δ*E*_ele_), and the salvation contribution (Δ*E*_sol_).

### Confocal Microscopy

J774 macrophages were seeded on 13-mm cover slips and incubated with bacteria at moi = 2.5 with or without curcumin for 0.5, 3, or 5 h. The extracellular bacteria were killed with 10 µg/ml of gentamicin at 0.5 h after infection. The samples were fixed with 4% paraformaldehyde at 4°C for 0.5 h at each time point. Permeabilization and blocking were performed with 0.1% Triton X-100 and 5% BSA, and the samples were further incubated with a rabbit antibody (Abcam) against *L. monocytogenes* for 2 h at room temperature as the primary antibody and an Alexa Fluor 594-conjugated chicken antibody (Molecular Probes) for 1 h at room temperature as the secondary antibody to indicate the bacteria, while F-actin was stained with phalloidin coupled to Alexa 488 (Molecular Probes).

For the bacterial Live/Dead assay, J774 cells were grown in 100-mm dishes (Thermo Fisher Scientific, USA) at a density of 3.0 × 10^6^ cells/dish overnight. The cells were treated with or without curcumin and infected with *L. monocytogenes* at moi = 2.5 for 5 h. At 0.5 h after infection, the extracellular bacteria were killed with 10 µg/ml of gentamicin. The cells were directly lyzed with PBS mixed with 0.2% Triton X-100 for 2 min at room temperature. The mixtures were centrifuged at 1,000 *g* for 10 min, and the supernatants were centrifuged at 10,000 × *g* for a further 20 min at 4°C. The viability of the bacteria in these cells was determined using the LIVE/DEAD^®^ BacLight™ Bacterial Viability Kit (L13152) according to the manufacturer’s instructions.

The therapeutic effect of curcumin on cell survival, which was mediated by LLO or mutations, was also assessed by using the LIVE/DEAD (green/red) reagent (Roche) according to the manufacturer’s instructions.

All the samples were observed under a confocal laser scanning microscope (Olympus, Tokyo, Japan).

### Electron Microscopic Observation

J774 cells were grown in 100-mm dishes at a density of 3.0 × 10^6^ cells/dish overnight. The cells were infected with bacteria at moi = 2.5, and curcumin was added at a 16 µg/ml concentration. At 0.5 h after infection, the extracellular bacteria were killed by gentamicin. At various times after infection, the samples were collected and examined with an electron microscope according to previous studies (18).

### Antibodies and Membrane-Binding Assay

A membrane-binding assay was conducted to determine whether curcumin had an effect on protein binding to target membranes. Sheep erythrocytes were lyzed in 20 mM MgCl_2_, broken by sonication, and then, centrifuged at 3,000 × *g* for 10 min, and the supernatants were centrifuged at 20,000 × *g* for 2 h. Precipitate was added to the mixture containing pre-incubated LLO or mutants with various concentrations of curcumin for 30 min, following incubation for a further 15 min.

A total of 10 µl of each sample was loaded onto a 12% SDS-PAGE after boiling in Laemmli sample buffer. The proteins were transferred to polyvinylidene fluoride (PVDF) membranes (Wako Pure Chemical Industries Ltd., Osaka, Japan) and blocked with 5% non-fat dried milk at room temperature for 2 h. To test the LLO expression, a rabbit antibody reactive to LLO (Abcam) was diluted at 1:2,000 and applied for 2 h for use as the primary antibody, and a horseradish peroxidase-conjugated antibody (Proteintech) at 1:3,000 was applied for another 2 h as the secondary antibody. Beta-actin (Proteintech) was used as an internal control in this assay, and it was used according to the recommended dilution.

The blots were detected using Amersham ECL Western blotting detection reagents (GE Healthcare, Buckinghamshire, UK).

### Expression of Lysosome-Associated Membrane Protein-1 (LAMP-1)

J774 cells were plated in 100-mm dishes at a density of 3.0 × 10^6^ cells/dish overnight. The cells were infected with bacteria at moi = 2.5, and curcumin was added at a concentration of 16 µg/ml. At 0.5 h after infection, the extracellular bacteria were killed by gentamicin. The expression of LAMP-1 protein at 3 and 5 h after infection was detected according to the instructions recommended by the manufacturer (Proteintech).

### Oligomerization Assay

Listeriolysin O and its mutated forms were pre-incubated with curcumin at 37°C, and protein oligomerization was induced *in vitro*; the protocol was performed as previously described (11).

### Animal Experiments

For all the following animal experiments, *L. monocytogenes* cultures were grown in TSB until reaching an OD_600_ = 0.8 and resuspended in PBS, following the use of PBS to adjust the bacterial concentration for different studies. Additionally, curcumin-treated mice were given 200 mg/kg curcumin subcutaneously at 2 h after infection and then at 8-h intervals until reaching defined time points.

Suspensions of 4 × 10^6^ colony-forming units (CFUs) of bacteria were injected for mortality studies. The mice that were injected with PBS were used as the control group. The mortality analysis was monitored after 96 h. For other animal experiments, at the indicated times after infection, the mice were killed by cervical dislocation to avoid pain. Samples containing 1 × 10^6^ CFUs of bacteria were injected for the bacterial loading assay and pathological analysis, and the data analysis was calculated by weighing and homogenizing the livers and spleens 48 h after infection. The organs were placed in 1% formalin for pathological analysis and stained with hematoxylin and eosin and then observed with a light microscope. The mice were injected with 4 × 10^5^ CFUs of bacteria to determine the potential effect of curcumin on bacterial growth and clearance, and the mice were sacrificed 3 or 7 days postinfection, and their spleens and livers were homogenized in PBS; the bacterial load was calculated as mentioned previously.

### Statistical Analysis

All the statistical analyses were performed using SPSS 13.0 software, defining differences to non-curcumin-treated groups as significant (*P < 0.05 and **P < 0.01).

## Results

### Curcumin Antagonizes the Hemolytic Activity of LLO and Protects Cell from the Cytotoxicity Induced by *L. monocytogenes*

**Figure 1:**
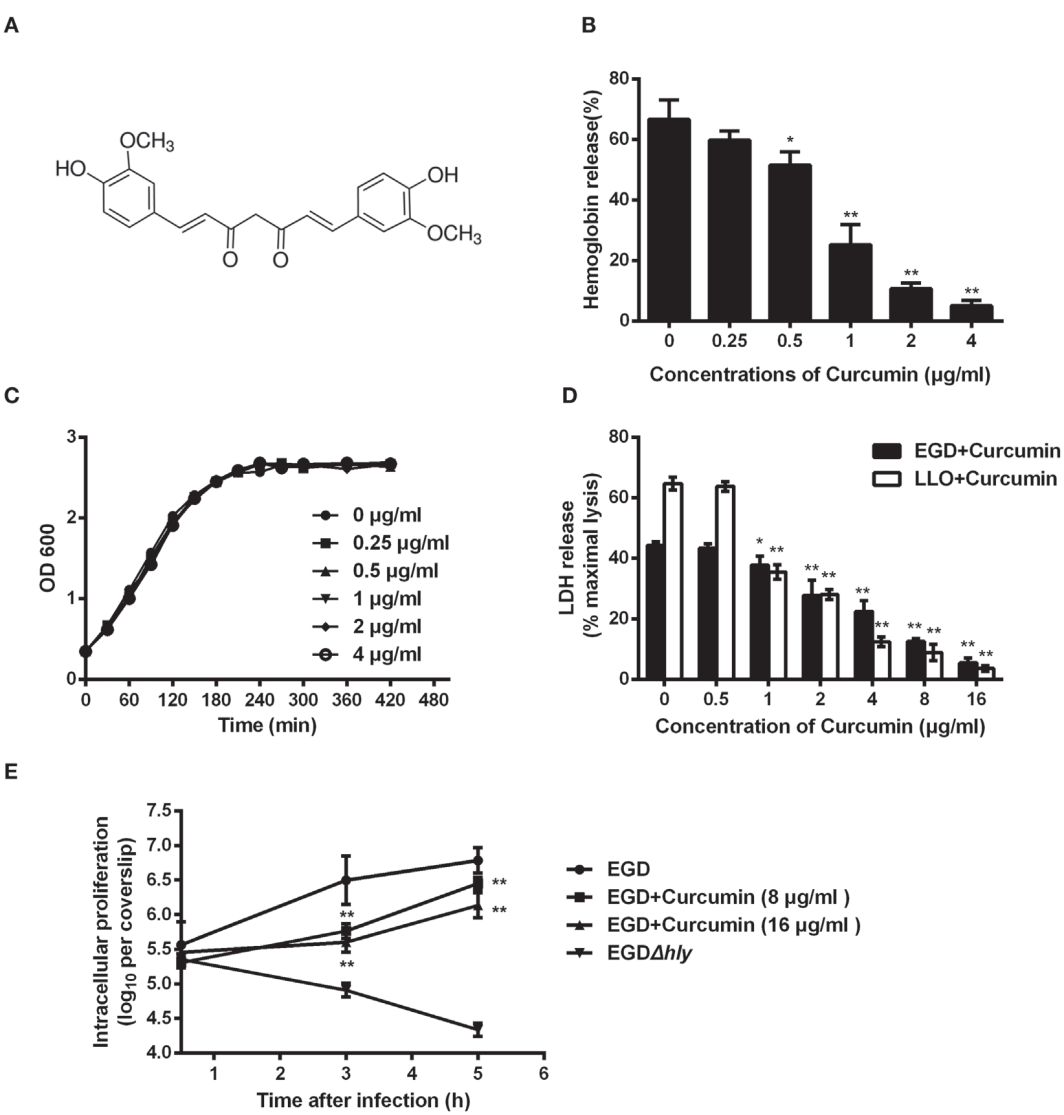
**Curcumin inhibits *Listeria monocytogenes* (*L. monocytogenes*)-induced cytotoxicity by suppressing the hemolysis of listeriolysin O (LLO)**. **(A)** The chemical structure of curcumin. **(B)** Hemolytic activity of LLO pre-incubated with or without curcumin. The hemoglobin release is shown as a function of curcumin-antagonized, LLO-mediated hemolytic activity. **(C)** Growth curves for the *L. monocytogenes* strain EGD (WT) cocultured with different concentrations of curcumin. Similar results were obtained in two independent experiments. **(D)** The cytotoxicity of EGD and LLO on J774 cells in the presence of curcumin. The cytotoxicity was determined by relative lactate dehydrogenase (LDH) release 5 h after infection. **(E)** Time-course infection dynamics of EGD or EGDΔ*hly* after treatment with curcumin (8 or 16 µg/ml). The total colony-forming units (CFUs) of intracellular bacteria were determined by viable count measurements. In panels **(B,D,E)**, mean ± SD values for three independent experiments are shown. *P* values were calculated using one-way analysis of variance (ANOVA) (*P < 0.05 and **P < 0.01).

Previous studies in our lab demonstrated that some natural flavonoids with similar structures possess different inhibitory effects on the hemolytic activity of LLO by sharing similar mechanisms (19). Here, we found that curcumin (Figure 1A), a polyphenol, could also inhibit LLO-induced hemolysis. The result of the hemolysis assay revealed that the lysis of sheep red blood cells was significantly inhibited (*P* < 0.05) when curcumin was added at the indicated concentration of 0.5 µg/ml (Figure 1B). This result suggested that the significant reduction in the hemolytic activity of LLO probably occurred through a direct interaction of curcumin with this toxin and effectively antagonized its activity. Consistent with this finding, cocultured *L. monocytogenes* with curcumin at the indicated concentrations was enough to inhibit the hemolytic activity of LLO, and they had almost no effect on bacterial growth (Figure 1C). Taken together, curcumin at 0.5 µg/ml is sufficient to decrease the hemolytic activity of LLO, while it has almost no effect on the phagocytosis of *L. monocytogenes* under experimental conditions. Curcumin does not belong to the flavonoid family, and its structure was not similar to that of these compounds. Thus, we hypothesized that curcumin antagonizes LLO activity through a different mechanism.

Listeriolysin O is known as an essential virulence factor that mediates pathogen escape from the phagosome into the cytoplasm. We have shown the first evidence that curcumin could effectively inhibit the hemolytic activity mediated by LLO, and therefore, we hypothesized that curcumin is capable of blocking the escape of *L. monocytogenes*, resulting in a decrease in cytotoxicity caused by *L. monocytogenes*. The result in Figure 1D shows that the cytotoxicity caused by *L. monocytogenes* was significantly inhibited by curcumin, as evaluated by the release of LDH. Compared with the untreated group, 2 µg/ml curcumin significantly (*P* < 0.01) decreased the LDH release caused by *L. monocytogenes*, and cell death was reduced from 44.32 to 27.82%, suggesting that curcumin could protect cell membranes from damage caused by *L. monocytogenes*. Furthermore, to demonstrate that this protective effect is highly associated with curcumin, decreasing the hemolytic activity of LLO, we incubated LLO with different concentrations of curcumin, which were subsequently cocultured with J774 cells for 5 h; as expected, the cytotoxicity depended on the LLO. This result indicates that curcumin treatment reduced the *L. monocytogenes-*induced LDH release, which was the consequence of decreasing LLO membrane perforation. In addition, we tested the impact of curcumin on intracellular bacterial growth. As shown in Figure 1E, the *L. monocytogenes* lacking LLO (strain EGDΔ*hly*) display a defect in bacterial intracellular growth, and the wild-type strains (EGD) grow rapidly in J774 cells, while at the present curcumin concentration (16 µg/ml), the growth of wild-type bacteria was decreased by 87.47 and 77.62% at 3 and 5 h, respectively.

### Analysis of the Curcumin Binding Sites against LLO

**Figure 2:**
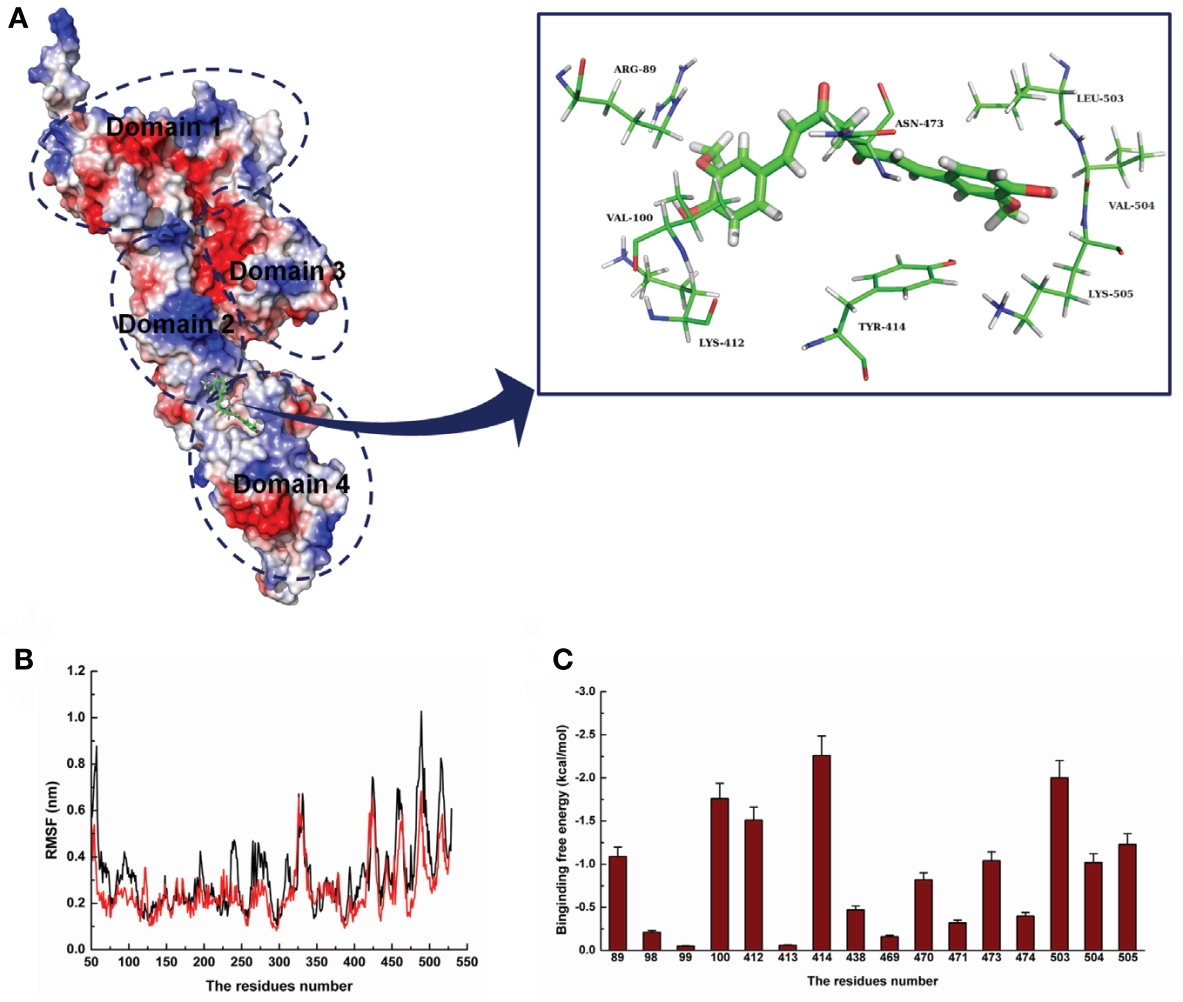
**The 3D structural determination of listeriolysin O (LLO) with a curcumin complex using molecular modeling method**. **(A)** The structure of LLO–curcumin. **(B)** The root-mean-square fluctuation (RMSF) displayed by the protein during MD simulations of LLO–curcumin is presented. **(C)** The decomposition of the binding energy on a per-residue basis in the binding sites of the LLO–curcumin complex.

For a stable LLO–curcumin structure, the standard molecular dynamics simulations were performed for the complex. As shown in Figure 2A, the stable structure of LLO with curcumin was given by the 200-ns MD simulation. It is shown that curcumin could be exactly embedded into the split between domains 2 and 4 in LLO via hydrophobic interactions. In detail, it is clear that the benzene ring on the left side of curcumin can form a strong interaction with Arg89 and Val100, which plays an important role in stabilizing the left side of curcumin. Moreover, Leu503, Val504, and Lys505 can also form a strong interaction with the right side of curcumin. In addition, the plane of the benzene ring on the right side of curcumin is parallel to the plane of the benzene ring in residue Tyr414. Then, a strong π–π interaction between this residue and curcumin most likely exists, leading to the stability of the right side of curcumin with LLO.

To validate the LLO–curcumin binding sites, the root-mean-square fluctuation (RMSF) of the residues around the LLO binding sites in the complex and free protein was calculated to explore the flexibility of these residues. As shown in Figure 2B, the flexibilities in the LLO binding sites in the presence and absence of curcumin are clearly different. The residues (80–100, 400–510) in the LLO binding sites that bind with curcumin show a small degree of flexibility with RMSF of <0.4 nm when compared with free protein, indicating that these residues seem to be more rigid as a result of their binding to curcumin. Through the above information, it was initially decided that the stabilization at the LLO–curcumin binding site was mostly due to residues Arg89, Val100, Lys412, Tyr414, Leu503, Val504, and Lys505, as shown in Figure 2A.

To confirm the LLO–curcumin binding sites, the contribution of the residues to the binding energy between curcumin and LLO was calculated using the MM-PBSA method. As shown in Figure 2C, Arg89, Val100, and Lys412 have strong appreciable binding energy contributions with values of <−1.0 kcal/mol, indicating the strong interaction between the LLO and the left part of the curcumin. Consistent with the results of the above analysis, Tyr414, Leu503, Val504, and Lys505 have strong interactions with curcumin, with values of ~−2.26, ~−2.00, ~−1.02, and ~−1.23 kcal/mol, respectively. In summation, it is verified that the key residues of the binding sites are Arg89, Val100, Lys412, Tyr414, Leu503, Val504, and Lys505.

To confirm the binding site in the LLO–curcumin complex, the same process of MD simulations was performed for the complex systems involving V100A-LLO and L503A-LLO mutants with curcumin, and the binding free energies of the two complexes were then calculated by using the MM-PBSA method. Subsequently, the binding free energies of curcumin with the two mutants were measured by the fluorescence spectroscopy quenching method. The total binding free energy of WT-LLO, V100A-LLO, and L503A-LLO with curcumin complexes and their detailed energy contributions are summarized in Table 1. The calculations of the binding free energy for the complexes revealed that the binding free energies of the mutants showed a decrease compared with the WT-LLO with the curcumin complex. However, according to the results from the fluorescence spectroscopy quenching method, the binding free energy between curcumin and the protein decreases in the following order: WT > V100A-LLO > L503A-LLO, which is consistent with the results of calculations based on the MD simulation. Thus, it is clear that the binding site of the LLO–curcumin complex is due to the residues Arg89, Val100, Lys412, Tyr414, Leu503, Val504, and Lys505.

**Table 1:** **The binding free energy (kcal/mol) of WT-LLO, V100A-LLO, and L503A-LLO systems based on the computational method and the values of the binding constants (*K*_A_) based on the fluorescence spectroscopy quenching**.

Proteins	WT-LLO	V100A	L503A
Binding energy	−8.8 ± 1.1	−5.4 ± 0.9	−4.7 ± 1.2
*K*_A_ (1 × 10^4^), L⋅mol^−1^	5.5 ± 1.5	4.7 ± 1.1	3.8 ± 0.9

### Principal Component Analysis (PCA) of the Movement of LLO from the Complex

**Figure 3:**
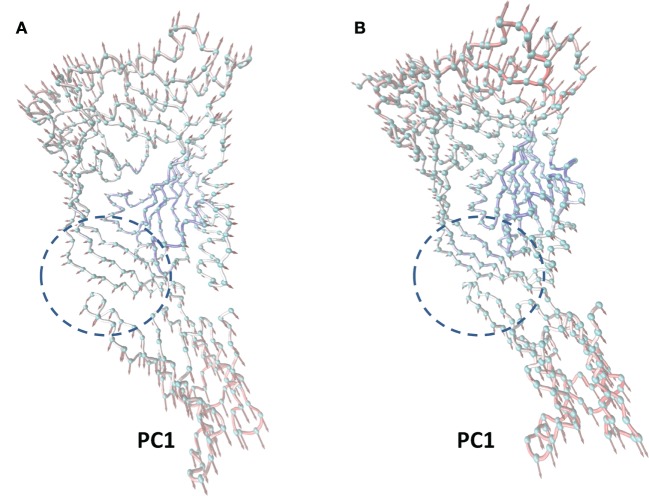
**The principal component analysis (PCA) of motion for listeriolysin O (LLO)**. The first principal component (PC1) in free protein **(A)** and the first PC1 in the complex **(B)** obtained by PCA are depicted by cones on the alpha carbon atoms. The length of the cones represents the magnitude of the motion. The dotted line range represents the curcumin–LLO binding region.

In this work, the most significant motions of the protein in a complex or an unliganded state were identified to explore the inhibition mechanism of curcumin by PCA on the basis of the MD trajectory of the free LLO and the LLO–curcumin complex. As shown in Figure 3A, there is an extended motion between domain 2 and domain 4 to the entire conformation of the free protein in the first principal component (PC1) (as represented by the dotted line in Figure 3), which is large enough to meet the requirement of the conformational transition for LLO from the monomer to the oligomer. Interestingly, curcumin could bind exactly in the split between domain 2 and domain 4 of the LLO based on the MD simulation, indicating that the motion of domain 2 and domain 4 in LLO could be influenced by the binding of curcumin. As we had expected, the extended motion between domain 2 and domain 4 is obviously weaker than that of the unliganded LLO due to the binding of curcumin with the split between domain 2 and domain 4, as shown in Figure 3B. Then, the motion of the conformation transition for LLO from the monomer to the oligomer is confirmed to be restricted by the binding of curcumin with LLO.

In summary, based on these findings, the following inhibition mechanism was realized: the binding of curcumin to the split between domains 2 and 4 in LLO blocks the transition in the conformational change from the monomer to the oligomer for LLO, leading to a decrease in the lytic activity of LLO.

### Curcumin Decreases the Hemolytic Activity of LLO by Influencing Its Oligomerization

**Figure 4:**
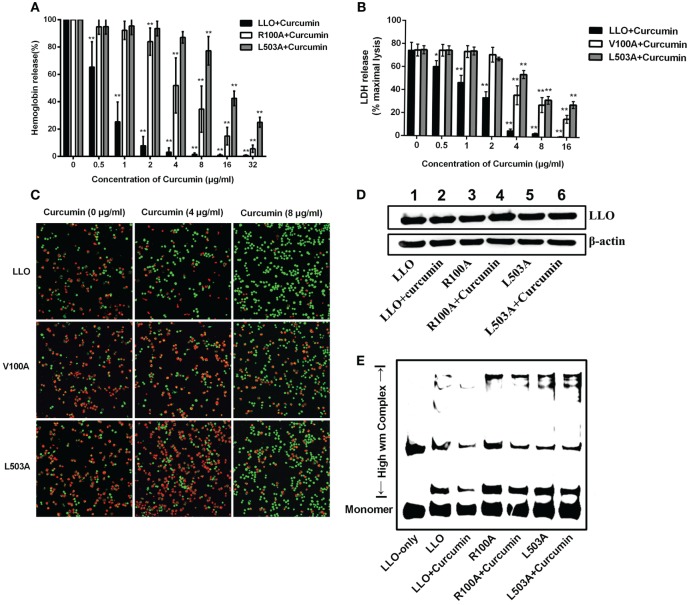
**Curcumin interferes with listeriolysin O (LLO) oligomerization by interacting with V100 and L503**. **(A)** Comparison of hemolytic activity relative to LLO, followed by pre-incubation with curcumin. **(B)** The cytotoxicity of two LLO mutants and LLO with various concentrations of curcumin. The lactate dehydrogenase (LDH) release was detected as described in Figure 1D. In panels **(A,B)**, mean ± SD values for three independent experiments are shown. *P* values were calculated using one-way analysis of variance (ANOVA) (*P < 0.05 and **P < 0.01). **(C)** Representative images of J774 cells that were challenged with LLO or mutants that co-incubated with curcumin. Cells with damaged membranes are shown in fluorescent red using ethidium homodimer-1 (EthD-1) and alternatively shown in fluorescent green using calcein-AM. **(D)** The impact of curcumin on the membrane-binding activity of wild-type LLO or the two mutants. The levels of the membrane-binding activity in different groups are visualized by Western blot, and the proteins of interest [LLO and β-actin (control)] were detected using specific antibodies. **(E)** The impact of curcumin on the oligomerization of LLO and two mutants. Oligomer formation was determined by Western blot using specific antibodies. In panels **(C**–**E)**, images are representative of three independent experiments.

Since V100 and L503 were two amino acids that were predicted by molecular dynamics simulation as the key potential binding sites, a hemolysis assay was first developed to validate the abovementioned results. The hemolytic assay showed that in comparison with WT-LLO, the protective effect of curcumin on LLO mutation-induced hemolysis in sheep blood cells significantly decreased by 8-fold and 16-fold in V100A and L503A (Figure 4A). Consistent with this result, the result from Figure 4B shows that compared with WT-LLO, the protective effects of curcumin on LLO mutations were 8- and 16-fold lower in V100A and L503A, respectively. Furthermore, the same results were observed in the live/dead and cytotoxicity assays, and the sensitivity of LLO to curcumin was more severely affected by the L503A mutation than the L100A mutation. Taken together, these results indicate that V100A and L503A were two important amino acids through which curcumin interfered with LLO (Figure 4C).

To express its cytolytic activity, the LLO monomers were first secreted by *L. monocytogenes*, subsequently binding to the target membrane, and then, oligomerization occurred to form pores and directly led to cytolytic activity. Thus, the influence of LLO and mutations on the membrane-binding assay was evaluated by Western blot assay, and no visual differences were detected between WT-LLO/V100A/L503A and the sample that was treated with curcumin, indicating that curcumin did not have an obvious impact on the binding of LLO/mutations to the target membrane (Figure 4D). Pretreating curcumin with LLO results in the significantly lower production of high molecular-weight LLO complexes, while this decreasing effect was less sensitive in LLO mutations, suggesting that this compound could directly reduce the oligomer formed by LLO (Figure 4E). Taken as a whole and consistent with the predictions derived from the molecular modeling, these observations demonstrated that curcumin could decrease the activity of LLO by interfering with the oligomer formation of LLO.

### Curcumin Reduces *Listeria* Growth in the Macrophage Cell Line (J774) by Interfering with LLO-Dependent Bacterial Phagosomal Escape

**Figure 5:**
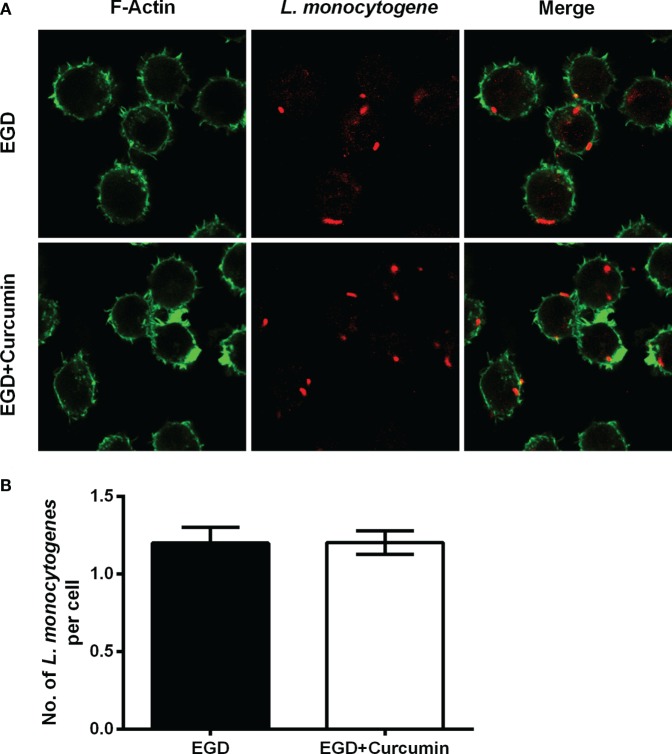
**Curcumin has no significant influence on phagocytosis in macrophages**. **(A)** The representative images of cells that were infected with EGD for 30 min at moi = 2.5 in the presence or absence of curcumin. Images are representative of three independent experiments. *Listeria monocytogenes* (*L. monocytogenes*) stained red (rabbit anti-*L. monocytogenes* and Alexa Fluor 594-conjugated chicken anti-rabbit), and F-actin stained green (Alexa Fluor 488 phalloidin). **(B)** The number of intracellular bacteria per cell. The number of bacteria was automatically calculated 30 min after infection. Mean ± SD values for three independent experiments are shown (*n* = 200). *P* value was calculated using two-tailed Student’s *t*-test.

**Figure 6:**
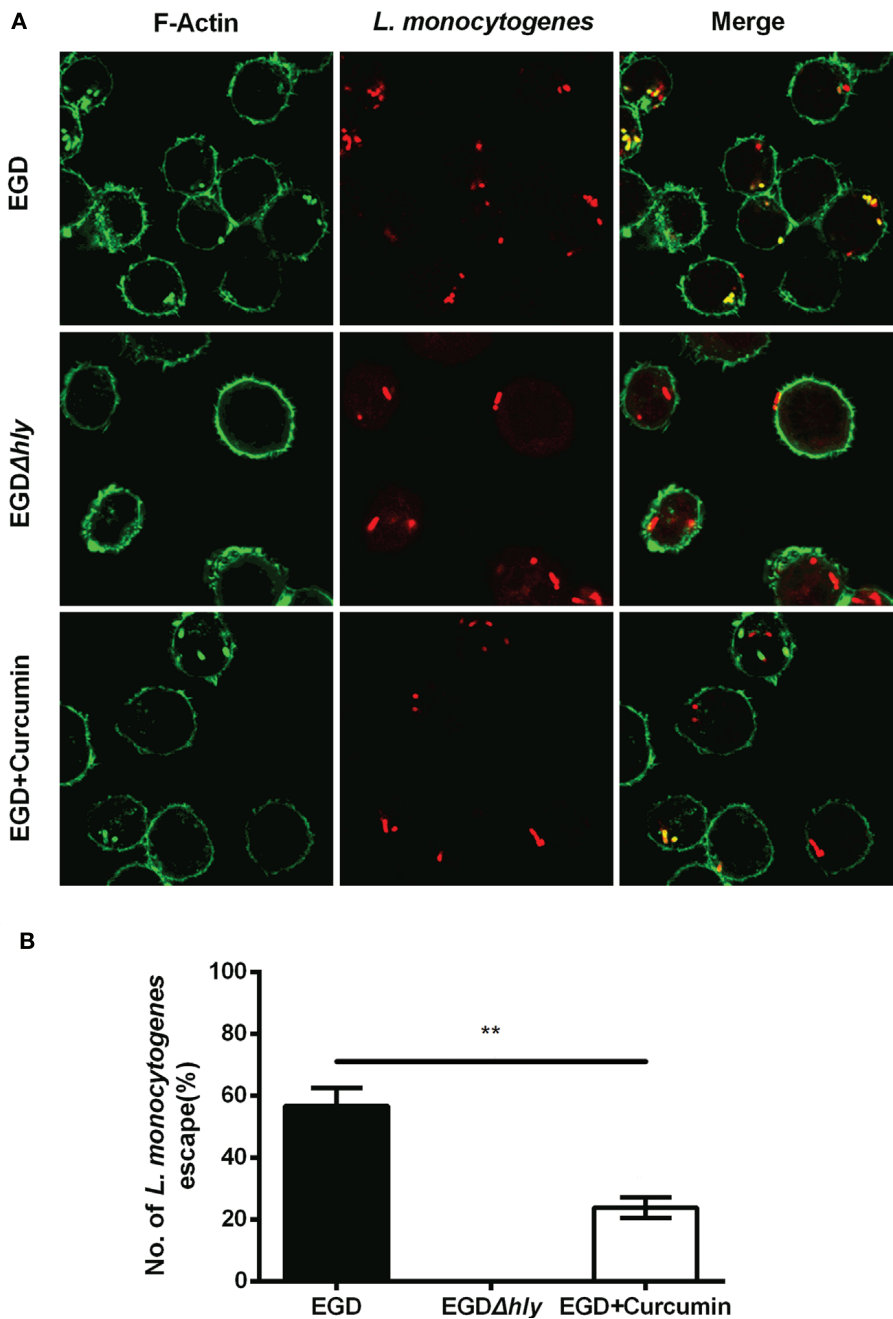
**Curcumin inhibits *Listeria monocytogenes* (*L. monocytogenes*)-induced actin polymerization**. **(A)** Representative images of J774 cells infected with EGD or EGD*Δhly* in the presence or absence of curcumin at 3 h after infection. Images are representative of three independent experiments. Bacteria are labeled in red, and F-actin is labeled in green as described in Figure 5A. **(B)** The number of escaping *L. monocytogenes*. The number of escaping bacteria (stained yellow) was automatically calculated. Mean ± SD values for three independent experiments are shown (*n* = 200). *P* value was calculated using two-tailed Student’s *t*-test (**P < 0.01).

To gain insight into the mechanism through which curcumin prevents *L. monocytogenes* growth in J774 cells and clearly demonstrates which step of the intracellular life cycle was impacted, the cells were incubated with curcumin when infected with EGD or EGDΔ*hly* at the indicated time points (0.5, 3, and 5 h), washed, and fixed. The bacteria were labeled with red immunostaining, and F-actin was labeled with green immunostaining. The result at 0.5 h showed that no significant differences were observed between the EGD group or the curcumin-treated group (Figures 5A,B), suggesting that the concentration of curcumin (16 µg/ml) that inhibited intracellular bacterial growth has no significant influence on phagocytosis. Bacterial polymerization of actin is a typical feature associated with bacterial escape from the phagosome for inducing bacterial motility. We have demonstrated that curcumin could antagonize the hemolytic activity of LLO, suggesting that curcumin was able to limit the number of bacteria that would escape. As we expected, at 3 h after infection, and with EGDΔ*hly* as the negative control, the results were consistent with earlier studies showing that the LLO-deficient strains remained trapped in the phagosome and were incapable of recruiting actin. By contrast, ~56.71% of wild-type strains (yellow; merge) were labeled with both bacterial fluorescent antibody and F-actin fluorescent antibody (Figure 6A). The percentage of bacteria escaping into the cytoplasm and acquiring an actin tail was 23.94%, and it was strikingly lower in curcumin-treated macrophages (Figure 6B). Taken together, these results demonstrated that curcumin effectively reduced bacterial multiplication by limiting the pathogen’s LLO-mediated phagosomal escape.

**Figure 7:**
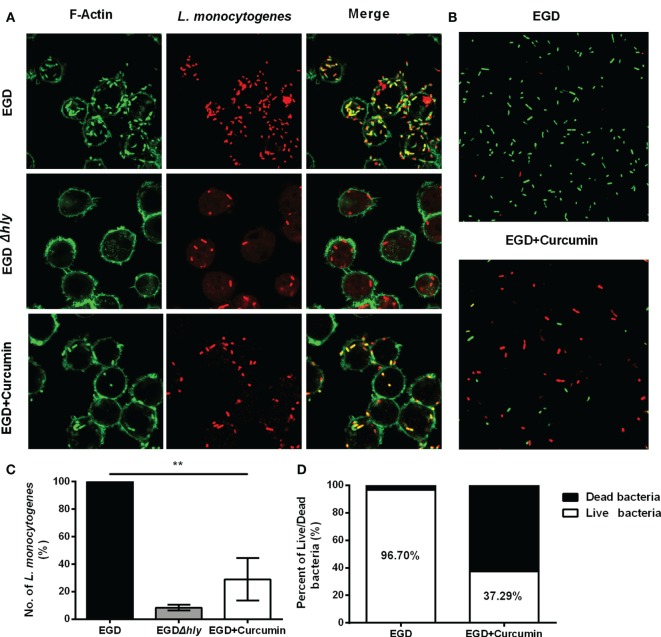
**Curcumin decreases the intracellular replication of *Listeria monocytogenes* (*L. monocytogenes*)**. **(A)** Representative images of J774 cells infected with EGD or EGD*Δhly* in the presence or absence of curcumin at 5 h after infection. Images are representative of three independent experiments. *L. monocytogenes* was stained red using *Listeria*-specific antibody (primary antibody) and Alexa Fluor 594-conjugated chicken antibody (secondary antibody). F-Actin was stained green using phalloidin coupled to Alexa 488. **(B)** The number of *L. monocytogenes* bacteria per cell. The EGD-treatment group was arbitrarily set as 100% as the WT treatment group. Mean ± SD values for three independent experiments are shown (*n* = 200). *P* value was calculated using two-tailed Student’s *t*-test (**P < 0.01). **(C)** The impact of curcumin on the bactericidal activity of macrophages. Images are representative of three independent experiments. Intracellular bacteria were exposed to SYTO 9 (green; live bacteria) and propidium iodide (red; dead bacteria). **(D)** The percentage of live bacteria relative to dead bacteria was automatically calculated for 500 bacteria. Similar results were obtained in two independent experiments.

Furthermore, intracellular bacterial replication was measured by immune staining for 5 h after infection (Figure 7A). In the macrophages that were infected with EGD, abundant amounts of bacteria were replicated and stained positive for F-actin, and this group served as the positive control (100% bacteria), whereas LLO-deficient *L. monocytogenes* remained confined in the phagosome with few bacteria in the cell (~8.39%). Treating the bacteria with 16 µg/ml resulted in a decreased percentage from 100 to 29.03 (Figure 7B). To assess whether the bacteria were alive or dead in the macrophages 5 h after infection, intercellular bacteria were collected and stained with PI or SYTO 9 (Figure 7C). The number of live bacteria in the curcumin-treated group decreased from 96.7 to 37.29% (Figure 7D), indicating that treating the macrophages with curcumin helped the cells to clear intracellular *L. monocytogenes*. Taken together, the results shown previously demonstrate that 16 µg/ml curcumin has no significant influence on phagocytosis but does inhibit the escape of *L. monocytogenes* by decreasing the LLO-mediated phagosome membrane perforation.

### Curcumin Effectively Protects Mice from *L. monocytogenes* Infection

**Figure 8:**
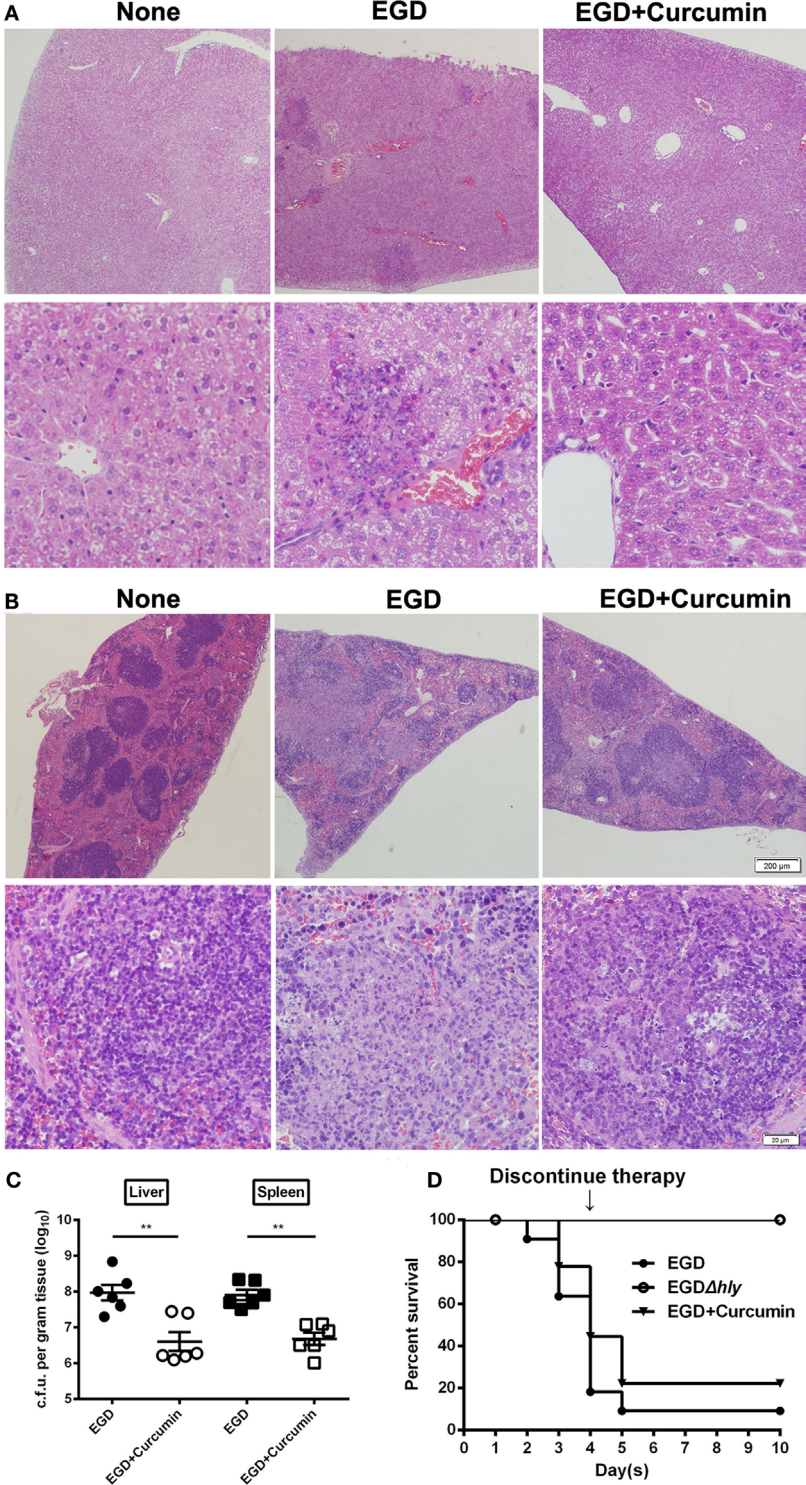
**Curcumin protects mice against *Listeria monocytogenes* (*L. monocytogenes*) infection**. Histopathological analyses of livers **(A)** and spleens **(B)** in untreated mice infected with EGD and curcumin-treated mice were determined 48 h after infection. The images are from a representative stained section (original magnification, ×40 and ×200, respectively). **(C)** The bacterial burden in the livers and spleens was determined 48 h after infection in the control group (non-curcumin-treated group) and the curcumin-treated group. Data are expressed as the mean ± SEM. *P* values were calculated using one-tailed Mann–Whitney test (**P < 0.01). **(D)** Survival curves for mice. The survival rates for EGD (●), EGD*Δhly* (○), and curcumin-treated (▾) mice after injection of 4 × 10^6^ colony-forming units (CFUs) of bacteria/mouse are shown. The survival rate was assessed every day for 10 days. Each experimental group consisted of 6 **(A–C)** or 10 (**D**) mice. Similar results in **(A**–**C)** were obtained in two independent experiments.

We then investigated whether targeting the LLO with curcumin could be an effective strategy for combating *L. monocytogenes* infections in the mouse model. Following an intraperitoneal administration of a sublethal dose of *L. monocytogenes* and the subcutaneous administration of curcumin at 200 mg/kg every 8 h for 48 h, the results showed that in the control group that was infected with bacteria, numerous spotty necroses were accompanied by inflammatory foci, congestion, and cell depletion as observed in the liver (Figure 8A), and a mass involving lymphocyte destruction with necrosis and congestion in the germinal centers of the spleen was also observed in the spleen (Figure 8B). In addition, the histopathology damage caused by *L. monocytogenes* in the liver and spleen was discernibly alleviated in the curcumin-treated group. Curcumin-treated mice displayed mild minor inflammatory lesions in their spleens and livers (Figures 8A,B). Curcumin has been shown to reduce bacterial burdens and mild histopathology damage during infection; the decreased bacterial load in the targeted tissues represented a significant (*P* < 0.01) reduction (Figure 8C). Moreover, with an intravenous inoculation of a lethal *L. monocytogenes* dose, the mice were partially protected, with 55.6% surviving compared to 80% dead controls 4 days after infection. Furthermore, curcumin-treated mice with 30% mice survival showed significantly longer survival times than the untreated group since therapy was discontinued. Altogether, these results suggest that after curcumin treatment, the mouse animal models were conferred with significant and effective protection against *L. monocytogenes* infection (Figure 8D). Together, our results established that the curcumin treatment systematically protected the mice from lethal infections of *L. monocytogenes*.

### Curcumin Facilitates *L. monocytogenes* Clearance by Counteracting Bacteria-Induced Phagosomal Escape

**Figure 9:**
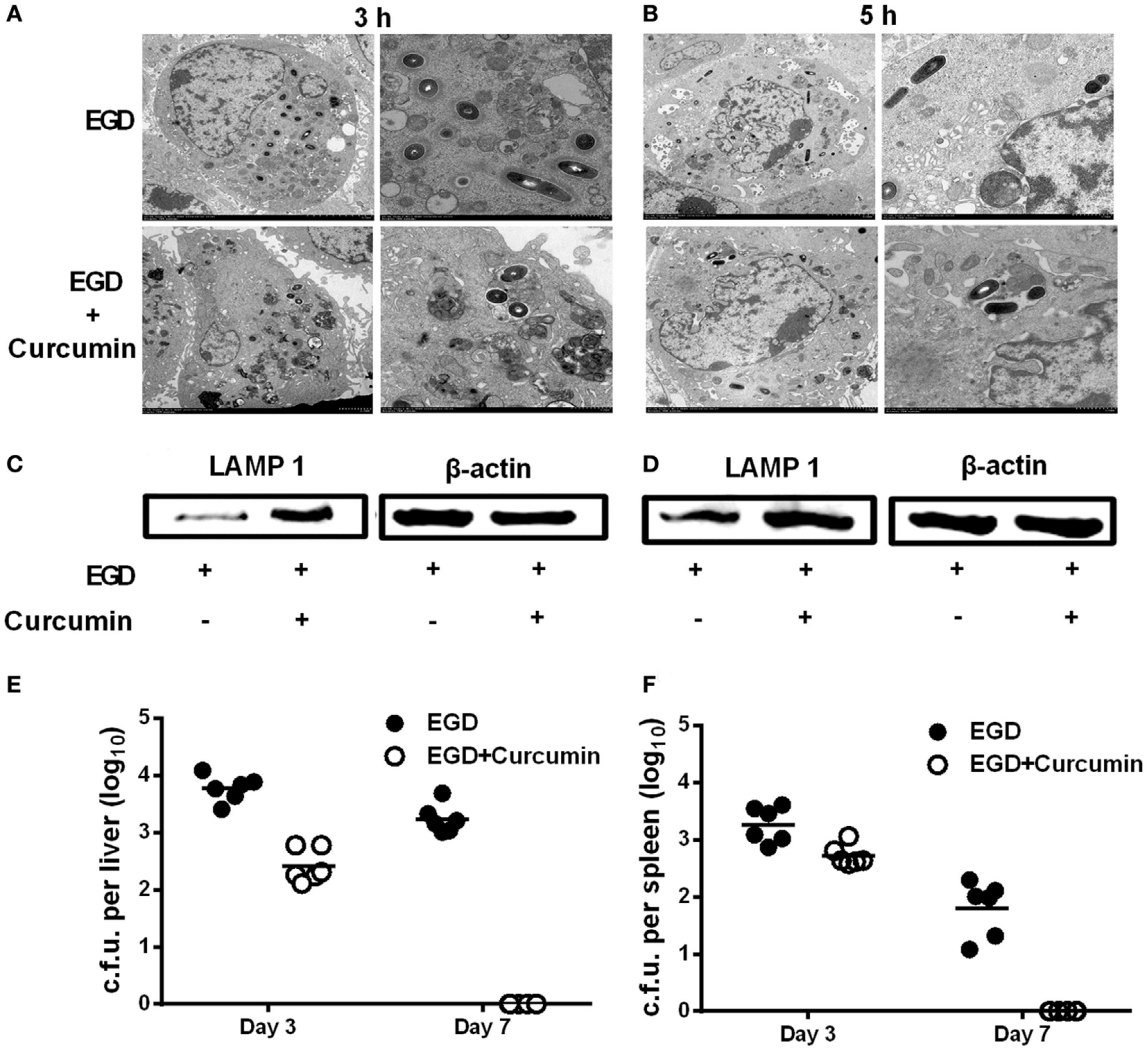
**Curcumin promotes the clearance of *Listeria monocytogenes* (*L. monocytogenes*) in macrophages and in mice**. J774 cells were infected with EGD with or without curcumin for 3 h **(A)** and 5 h **(B)** after infection, and images were analyzed by transmission electron microscopy (TEM) at different magnifications (×1,200 and ×3,000, respectively). Lysosome-associated membrane protein-1 expression at 3 h **(C)** and 5 h **(D)** after infection with EGD was visualized by Western blot. On days 3 and 7 after inoculation [4 × 10^5^ colony-forming units (CFUs) of EGD], quantification of viable bacteria in liver **(E)** and spleen **(F)** from BALB/c mice (*n* = 6) was determined. Images in **(A**–**D)** are representative of three independent experiments.

It is well demonstrated that LLO grants wild-type *L. monocytogenes* the ability to escape before phagosomes fuse with the lysosome, which is an important step for macrophages to participate in the degradation of pathogens within phagosomes. Thus, in order to examine whether curcumin could aid macrophages in clearing bacteria by targeting LLO, electron microscopic observation was performed, and the expression level of LAMP-1 was evaluated. Transmission electron microscopy observations were performed at 3 h (Figure 9A) and 5 h (Figure 9B) after the infection of J774 cells by *L. monocytogenes* EGD. The results demonstrated that after curcumin treatment, most invading bacteria remained surrounded by an intact phagocytic membrane. In addition, unlike the EGD strain, the phagosome membrane was destroyed and bacteria spread intracellularly after 3 and 5 h, and *L. monocytogenes* were often found within lysosomal bodies, indicating that curcumin could help the phagolysosome to clear the EGD while reducing LLO activity and limiting the bacteria in the endosomal membrane. Since LAMP-1 is the marker that can be detected in the lysosome membrane, LLO-deficient bacteria have been shown to remain trapped in LAMP-1-positive phagosomes. Thus, by using the Western blot assay, we evaluated LAMP-1 protein expression in the curcumin treatment group and the wild-type group. The results in Figures 9C,D show that more LAMP-1 protein expression was observed in the curcumin treatment group at both 3 and 5 h after infection, which suggested that more bacteria were confined with LAMP-1-positive J774 cells after curcumin treatment. This compound could facilitate the engulfment of more bacteria within the lysosome. Moreover, infection with a sublethal dose of *L. monocytogenes* is sufficient to trigger the immune system to clear the bacteria (8). Thus, we simultaneously investigated *L. monocytogenes* clearance in mice by establishing an *in vivo* model of intraperitoneal injection with a lower dose of bacteria. As shown in Figures 9E,F, mice infected with *L. monocytogenes* displayed a large number of bacterial colonies in their livers and spleens 3 days after infection, while the bacteria were still not cleared on day 7. Compared with this group, the curcumin-treated mice reflected higher resistance to bacterial infections because no *Listeria* colonies were detected in the spleens and livers on day 7, suggesting that curcumin effectively facilitates bacterial clearance depending on the host immune system.

## Discussion

*Listeria monocytogenes* is a facultative saprophytic bacterial pathogen that causes a high-mortality disease (30% mortality globally) called listeriosis (20, 21). Current clinical treatments for this pathogen largely rely on high doses of antibiotics, such as penicillin and gentamicin. In contrast to typical antibiotic-resistant bacteria, such as *Staphylococcus aureus*, most clinically isolated strains remain susceptible to these antibiotics (22). Due to limitations in antibiotic efficacy in terms of low outer membrane permeability in the host and due to intrinsic drawbacks of antibiotics (23, 24), *L. monocytogenes* infections have posed a severe challenge to public health (25, 26). To prevent *L. monocytogenes* from becoming another drug-resistant bacterium, or even worse, a new daunting bacterium equipped with multi-drug resistance in the post-antibiotic era, new therapeutic strategies are urgently needed to control *L. monocytogenes* infections.

Anti-virulence therapy has attracted great interest in recent years, and this strategy may afford an alternative choice that is effective against bacterial infections. Studies have shown that using natural compounds that decrease virulence activity could significantly affect intracellular infection with *L. monocytogenes* (27, 28). Moreover, most virulence factors are not essential for bacterial survival, suggesting that using this strategy might place bacteria under a milder pressure with lower chances of developing drug resistance. Supporting this idea, a previous study has demonstrated that after continuous exposure of *L. monocytogenes* to natural compounds, no development of drug-resistance strains was observed (29). Previous studies have demonstrated that LLO is critical for *L. monocytogenes* to escape from phagosomes, and LLO mutants do not cause any tissue damage or death in the mouse infection model, which renders this virulence an important drug target with which to combat *L. monocytogenes* infection (30–32).

In this study, we provide evidence that curcumin may be a potential anti-virulence agent that can be further developed against *L. monocytogenes* infection. *In vitro* studies showed that the effect of curcumin on LLO could clearly prevent *L. monocytogenes* from surviving in the cytoplasm and could reduce the colonization and toxicity of this bacterium in target cells, as well as facilitate the clearance of *L. monocytogenes* by macrophages. *In vivo* experiments suggested that curcumin could weaken the histopathological damage of the bacteria in an animal infection model, decreasing the mortality of the mice significantly. It has been shown that *L. monocytogenes* could be radically cleared by the host immune system after injection with a sublethal dose of bacterium (8). Consistent with this study, in the *in vitro* assay, compared with the untreated group, curcumin treatment could help the host to clear the same dose of bacteria more rapidly. Kohda et al. (33) showed that epigallocatechin gallate, the major tea catechin, inhibited the hemolytic activity of LLO, thereby inhibiting the escape of bacteria from the phagosome. Compared with that study, we performed more experiments to reveal the anti-virulence function of curcumin. Not only did we demonstrate that curcumin could decrease the LLO activity and restrict bacteria from escaping from phagolysosomes, we also showed that curcumin could facilitate the clearing of this bacterium in macrophages and in an animal infection model. Moreover, to our surprise, the effect of curcumin on inhibiting pore formation was not specific for LLO. We found that at similar concentrations, curcumin also exerted powerful effects in terms of attenuating the activity of other toxins in the CDC family, such as SLY and PLY (data not shown), suggesting that curcumin is a potential candidate against pore-forming toxins in the CDC family.

Previous studies in our lab have identified natural flavonoids without antimicrobial activity that could decrease the hemolytic activity of LLO. These compounds had similar structures and shared the same mechanism, directly engaging with loops 2 and 3 in domain 4, suggesting that this structure may be useful for the development of LLO inhibitors (11, 19). The structure of curcumin is quite different from these compounds but shows a powerful anti-hemolytic effect, which may offer another base structure for the development of new drugs. The results showed that by standard MD simulations, curcumin could bind specifically with the split between domains 2 and 4 of LLO by making strong contact with Val100 and Leu503, which is quite different from the results of previous reports. Principal component analysis showed that on the basis of the dynamic trajectory analysis, it was predicted that the engagement of curcumin with LLO could cause a conformational change in domains 2 and 4 of LLO. In free protein, it is obligatory that the extended motion between domains 2 and 4 to the entire conformation of the free protein could sufficiently meet the requirement of a conformational transition for LLO from a monomer to an oligomer. However, in the complex system, the extended motion between domains 2 and 4 was restricted, leading to a block in the conformational transition for LLO from the monomer to the oligomer. Owing to the block, the lytic activity of LLO in the complex is lower than that of the free protein.

Curcumin is a food-grade additive, and *L. monocytogenes* has the highest fatality rate among food-borne pathogens. Therefore, it may be possible to add curcumin to food to prevent *L. monocytogenes* infection (34). Unfortunately, the bioavailability of curcumin is low because it has intrinsically low water solubility (35). Thus, further investigations are required to translate this research into clinical applications to prevent or treat *L. monocytogenes-*related infectious diseases.

## Ethics Statement

The 8-week-old BALB/c male mice used in this study were obtained from the Experimental Animal Center of Jilin University. All the animal experiments were approved by and conducted in accordance with the guidelines of the Animal Care and Use Committee of Jilin University. Moreover, all animal experiments were conducted according to the Regulations for the Administration of Affairs Concerning Experiment Animals.

## Author Contributions

XD, JW, and XZ conceived and designed the experiments. XZ, BZ, YC, SC, ZT, and GL performed the experiments. JW and XD contributed reagents/materials/analysis tools. XZ, JW, and XD wrote the paper.

## Conflict of Interest Statement

The authors declare that the research was conducted in the absence of any commercial or financial relationships that could be construed as a potential conflict of interest.
